# (−)-Crebanine

**DOI:** 10.1107/S1600536811001231

**Published:** 2011-01-15

**Authors:** Tanwawan Duangthongyou, Arthit Makarasen, Supanna Techasakul, Nitirat Chimnoi, Sutatip Siripaisarnpipat

**Affiliations:** a Center of Excellence in Functional Materials, Department of Chemistry, Faculty of Science, Kasetsart University, Bangkok 10930, Thailand; b Chulabhorn Research Institute,Vibhavadee-Rangsit Highway, Laksi, Bangkok 10210, Thailand

## Abstract

The asymmetric unit of the title compound [systematic name: 9,10-dimeth­oxy-7-methyl-6,7,7a,8-tetra­hydro-5*H*-benzo[*g*][1,3]benzodioxolo[6,5,4-*de*]quinoline], C_20_H_21_NO_4_, contains two independent mol­ecules with very similar bond lengths and angles. The crystal packing exhibits voids of 131 Å^3^.

## Related literature

For related structures, see: Israilov *et al.* (1980 ▶); Blanchfield *et al.* (2003 ▶). For the chemistry, pharmacology and traditional uses of the title compound, see; Montririttigri *et al.* (2008 ▶) and Semwal *et al.* (2010 ▶).
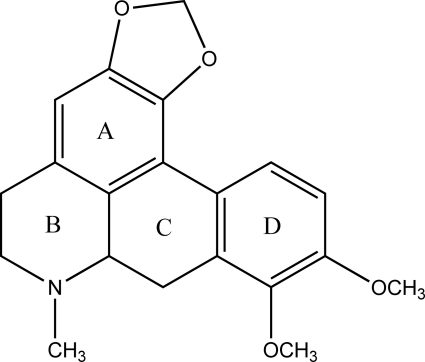


## Experimental

### 

#### Crystal data

C_20_H_21_NO_4_
*M* *_r_* = 339.38
Orthorhombic, 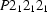

*a* = 4.4029 (3) Å
*b* = 20.5847 (15) Å
*c* = 39.612 (3) Å
*V* = 3590.2 (4) Å^3^
*Z* = 8
Mo *K*α radiation
μ = 0.09 mm^−1^
*T* = 298 K
0.22 × 0.16 × 0.12 mm

#### Data collection

Bruker SMART CCD area-detector diffractometer
31211 measured reflections
5054 independent reflections
3592 reflections with *I* > 2σ(*I*)
*R*_int_ = 0.071

#### Refinement

*R*[*F* ^2^ > 2σ(*F* ^2^)] = 0.105
*wR*(*F* ^2^) = 0.249
*S* = 1.2
5054 reflections
453 parameters
H-atom parameters constrained
Δρ_max_ = 0.55 e Å^−3^
Δρ_min_ = −0.25 e Å^−3^

### 

Data collection: *SMART* (Bruker, 2002 ▶); cell refinement: *SAINT* (Bruker, 2002 ▶); data reduction: *SAINT*; program(s) used to solve structure: *SHELXS97* (Sheldrick, 2008) ▶; program(s) used to refine structure: *SHELXL97* (Sheldrick, 2008) ▶; molecular graphics: *ORTEP-3* (Farrugia, 1997 ▶); software used to prepare material for publication: *SHELXL97* ▶.

## Supplementary Material

Crystal structure: contains datablocks global, I. DOI: 10.1107/S1600536811001231/hg2768sup1.cif

Structure factors: contains datablocks I. DOI: 10.1107/S1600536811001231/hg2768Isup2.hkl

Additional supplementary materials:  crystallographic information; 3D view; checkCIF report

## Figures and Tables

**Fig. 1.**
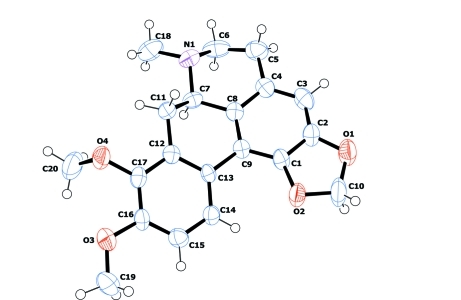
The structure of molecule 1 of crebanine showing 50% displacement ellipsoids.

**Fig. 2.**
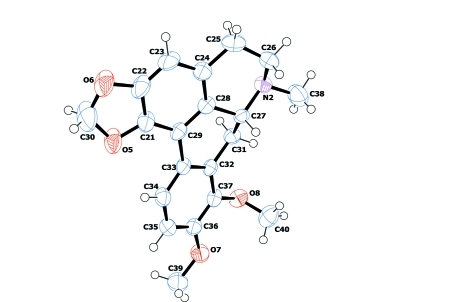
The structure of molecule 2 of crebanine showing 50% displacement ellipsoids.

## supplementary crystallographic information

### Comment

Crebanine is an aporphine alkaloid (Israilov *et al.*, 1980; Blanchfield *et al.*, 2003). It was isolated from the crude hexane extract of the dried tuber of Stephania venosa, which is native to Thailand and commonly used for treatment of variety of aliments under the local name "sabulead" (Montririttigri *et al.*, 2008; Semwal *et al.*,2010). *S. venosa* tuber and leaves were collected from Prachuabkirikhan province in the southern part of Thailand.

The asymmetric unit of the title compound [systematic name: 9,10-dimethoxy-7-methyl-6,7,7a,8-tetrahydro-5*H*-[1,3]dioxolo[4',5':4,5]benzo[1,2,3-de]benzo[*g*]quinoline], C_20_H_21_NO_4_, contains two independent molecules. There is very little difference between the bond lengths and angles of these molecules. The molecules are nearly planar (r.m.s deviation = 0.2894Å and 0.2413 Å). The molecule consists of four fused-rings (A, B, C and D). The six membered-rings B and C are both in distorted half-chair conformations. The porous crystal packing exhibits voids of 131 Å^3^. The structure is devoid of any classical hydrogen bonds.

### Experimental

Crebanine was isolated from the crude hexane extract of the dried tuber of *Stephania venosa*. After purification by classical liquid chromatography on silica gel and recrystallization from hexane-dichloromethane, crebanine was obtained as colorless needles, m.p. 115–116.5°C.

### Refinement

All H atoms were geometrically positioned and treated as riding atoms with distances C—H = 0.96 Å (CH_3_), 0.97 Å (CH_2_), 0.93 Å (CH), and *U*_iso_(H) = 1.20 *U*_eq_(C) for methylene and aromatic, 1.50 *U*_eq_(C) for methyl. The absolute structure could not be determined from the X-ray analysis, but it was known from earlier work on related compounds (Israilov *et al.*, 1980; Blanchfield *et al.*, 2003). 3,504 Friedel pairs were merged before the final refinement. The crystal structure contained solvent accessible voids of 131 Å^3 ^and showed no electrons in the voids.

### Crystal data

**Table:**

C_20_H_21_NO_4_	*F*(000) = 1440
*M**_r_* = 339.38	*D*_x_ = 1.256 Mg m^−^^3^
Orthorhombic, *P*2_1_2_1_2_1_	Mo *K*α radiation, λ = 0.71073 Å
Hall symbol: P 2ac 2ab	Cell parameters from 254 reflections
*a* = 4.4029 (3) Å	θ = 25–35°
*b* = 20.5847 (15) Å	µ = 0.09 mm^−^^1^
*c* = 39.612 (3) Å	*T* = 298 K
*V* = 3590.2 (4) Å^3^	Needle, colorless
*Z* = 8	0.22 × 0.16 × 0.12 mm

### Data collection

**Table:**

Bruker SMART CCD area-detector diffractometer	3592 reflections with *I* > 2σ(*I*)
Radiation source: Mo Kα	*R*_int_ = 0.071
graphite	θ_max_ = 28.3°, θ_min_ = 1.4°
φ and ω scans	*h* = −5→5
31211 measured reflections	*k* = −26→26
5054 independent reflections	*l* = −51→52

### Refinement

**Table:**

Refinement on *F*^2^	H-atom parameters constrained
Least-squares matrix: full	*w* = 1/[σ^2^(*F*_o_^2^) + (0.102*P*)^2^ + 1.8904*P*] where *P* = (*F*_o_^2^ + 2*F*_c_^2^)/3
*R*[*F*^2^ > 2σ(*F*^2^)] = 0.105	(Δ/σ)_max_ < 0.001
*wR*(*F*^2^) = 0.249	Δρ_max_ = 0.55 e Å^−^^3^
*S* = 1.2	Δρ_min_ = −0.25 e Å^−^^3^
5054 reflections	Extinction correction: *SHELXL97* (Sheldrick, 2008), Fc^*^=kFc[1+0.001xFc^2^λ^3^/sin(2θ)]^-1/4^
453 parameters	Extinction coefficient: 0.0011 (10)
0 restraints	

### Special details

**Table:**

Geometry. All e.s.d.'s (except the e.s.d. in the dihedral angle between two l.s. planes) are estimated using the full covariance matrix. The cell e.s.d.'s are taken into account individually in the estimation of e.s.d.'s in distances, angles and torsion angles; correlations between e.s.d.'s in cell parameters are only used when they are defined by crystal symmetry. An approximate (isotropic) treatment of cell e.s.d.'s is used for estimating e.s.d.'s involving l.s. planes.

### Fractional atomic coordinates and isotropic or equivalent isotropic displacement parameters (Å^2^)

**Table:**

	*x*	*y*	*z*	*U*_iso_*/*U*_eq_	
O1	−0.0157 (14)	−0.29778 (18)	0.91783 (10)	0.0651 (14)	
O2	0.2100 (12)	−0.22350 (17)	0.88249 (9)	0.0559 (12)	
O3	0.4781 (12)	0.06920 (18)	0.82737 (10)	0.0585 (12)	
O4	0.1040 (10)	0.09746 (18)	0.87877 (11)	0.0541 (11)	
O5	0.6133 (16)	1.07654 (18)	0.71607 (10)	0.0742 (17)	
O6	0.4220 (18)	1.14827 (19)	0.67722 (12)	0.089 (2)	
O7	0.9437 (13)	0.78611 (19)	0.77372 (10)	0.0602 (13)	
O8	0.5931 (10)	0.75386 (17)	0.72165 (10)	0.0479 (10)	
N1	−0.2107 (12)	−0.0207 (2)	0.98698 (12)	0.0476 (12)	
N2	0.2578 (11)	0.8658 (2)	0.61320 (11)	0.0434 (11)	
C1	0.1036 (14)	−0.1925 (2)	0.91134 (12)	0.0401 (13)	
C2	−0.0304 (15)	−0.2372 (2)	0.93191 (14)	0.0451 (14)	
C3	−0.1577 (16)	−0.2198 (3)	0.96193 (15)	0.0521 (16)	
H3A	−0.2482	−0.2506	0.9758	0.062*	
C4	−0.1484 (14)	−0.1539 (3)	0.97130 (14)	0.0448 (14)	
C5	−0.2810 (18)	−0.1329 (3)	1.00402 (14)	0.0589 (17)	
H5A	−0.225	−0.1638	1.0214	0.071*	
H5B	−0.5008	−0.1327	1.0022	0.071*	
C6	−0.1727 (17)	−0.0660 (3)	1.01402 (15)	0.065 (2)	
H6A	−0.2863	−0.0512	1.0335	0.078*	
H6B	0.0402	−0.068	1.0203	0.078*	
C7	−0.0060 (13)	−0.0364 (3)	0.95844 (12)	0.0386 (12)	
H7A	0.2024	−0.0261	0.9653	0.046*	
C8	−0.0189 (12)	−0.1080 (2)	0.94904 (13)	0.0364 (12)	
C9	0.1075 (13)	−0.1262 (2)	0.91788 (12)	0.0349 (12)	
C10	0.173 (2)	−0.2903 (3)	0.88925 (18)	0.082 (3)	
H10A	0.3693	−0.3101	0.8935	0.098*	
H10B	0.0819	−0.3117	0.8699	0.098*	
C11	−0.0825 (13)	0.0045 (2)	0.92741 (13)	0.0407 (13)	
H11A	−0.292	−0.0029	0.9209	0.049*	
H11B	−0.0597	0.0503	0.9328	0.049*	
C12	0.1226 (12)	−0.0127 (2)	0.89867 (13)	0.0353 (12)	
C13	0.2230 (12)	−0.0770 (2)	0.89452 (12)	0.0320 (11)	
C14	0.4221 (14)	−0.0913 (3)	0.86779 (13)	0.0398 (13)	
H14A	0.4952	−0.1333	0.8652	0.048*	
C15	0.5107 (15)	−0.0432 (3)	0.84522 (13)	0.0444 (14)	
H15A	0.6409	−0.0535	0.8275	0.053*	
C16	0.4091 (14)	0.0190 (2)	0.84870 (13)	0.0387 (13)	
C17	0.2161 (13)	0.0345 (2)	0.87532 (14)	0.0389 (12)	
C18	−0.158 (2)	0.0464 (3)	0.99844 (17)	0.077 (2)	
H18A	−0.2857	0.0555	1.0175	0.115*	
H18B	−0.2052	0.076	0.9805	0.115*	
H18C	0.0509	0.0514	1.0048	0.115*	
C19	0.674 (2)	0.0563 (3)	0.80014 (17)	0.070 (2)	
H19A	0.7034	0.0953	0.7872	0.105*	
H19B	0.5868	0.0234	0.786	0.105*	
H19C	0.8666	0.0415	0.8086	0.105*	
C20	0.319 (2)	0.1428 (3)	0.8904 (2)	0.083 (2)	
H20A	0.226	0.1849	0.8921	0.124*	
H20B	0.4864	0.1448	0.8749	0.124*	
H20C	0.3915	0.1298	0.9123	0.124*	
C21	0.5342 (18)	1.0426 (3)	0.68717 (13)	0.0518 (16)	
C22	0.413 (2)	1.0858 (3)	0.66409 (16)	0.064 (2)	
C23	0.3093 (19)	1.0661 (3)	0.63356 (16)	0.0607 (19)	
H23A	0.2322	1.0957	0.618	0.073*	
C24	0.3227 (15)	0.9997 (3)	0.62627 (14)	0.0482 (15)	
C25	0.2002 (17)	0.9756 (3)	0.59241 (15)	0.0595 (18)	
H25A	−0.02	0.976	0.593	0.071*	
H25B	0.2653	1.005	0.5747	0.071*	
C26	0.3056 (17)	0.9090 (3)	0.58427 (13)	0.0528 (16)	
H26A	0.1947	0.8926	0.5649	0.063*	
H26B	0.5197	0.91	0.5785	0.063*	
C27	0.4581 (12)	0.8834 (2)	0.64149 (12)	0.0350 (12)	
H27A	0.6677	0.8731	0.6351	0.042*	
C28	0.4386 (13)	0.9565 (2)	0.64921 (12)	0.0382 (13)	
C29	0.5500 (14)	0.9766 (2)	0.68092 (13)	0.0400 (13)	
C30	0.525 (4)	1.1408 (3)	0.7099 (2)	0.126 (5)	
H30A	0.366	1.153	0.7255	0.152*	
H30B	0.6971	1.1694	0.7136	0.152*	
C31	0.3784 (14)	0.8450 (2)	0.67290 (12)	0.0396 (13)	
H31A	0.1685	0.8532	0.679	0.048*	
H31B	0.4002	0.7989	0.6683	0.048*	
C32	0.5819 (13)	0.8637 (2)	0.70186 (12)	0.0357 (12)	
C33	0.6691 (13)	0.9292 (2)	0.70569 (12)	0.0367 (12)	
C34	0.8646 (16)	0.9456 (3)	0.73220 (13)	0.0463 (15)	
H34A	0.9318	0.9883	0.7343	0.056*	
C35	0.9600 (16)	0.8996 (3)	0.75542 (14)	0.0502 (15)	
H35A	1.0848	0.9116	0.7733	0.06*	
C36	0.8682 (15)	0.8351 (3)	0.75186 (13)	0.0440 (14)	
C37	0.6859 (14)	0.8179 (2)	0.72494 (13)	0.0382 (13)	
C38	0.310 (2)	0.7977 (3)	0.60256 (18)	0.075 (2)	
H38A	0.1769	0.7873	0.5841	0.112*	
H38B	0.2695	0.7691	0.6212	0.112*	
H38C	0.5171	0.7926	0.5955	0.112*	
C39	1.115 (2)	0.8034 (4)	0.80296 (16)	0.082 (3)	
H39A	1.1537	0.7652	0.8162	0.123*	
H39B	1.0024	0.8342	0.8162	0.123*	
H39C	1.3046	0.8224	0.7962	0.123*	
C40	0.8261 (19)	0.7140 (3)	0.7071 (2)	0.070 (2)	
H40A	0.7537	0.6701	0.7051	0.105*	
H40B	1.0026	0.7148	0.7213	0.105*	
H40C	0.8778	0.7303	0.6851	0.105*	

### Atomic displacement parameters (Å^2^)

**Table:**

	*U*^11^	*U*^22^	*U*^33^	*U*^12^	*U*^13^	*U*^23^
O1	0.103 (4)	0.039 (2)	0.053 (2)	−0.013 (3)	0.001 (3)	0.0042 (18)
O2	0.095 (4)	0.0325 (19)	0.040 (2)	−0.010 (2)	0.005 (2)	−0.0030 (16)
O3	0.075 (3)	0.043 (2)	0.057 (2)	−0.002 (2)	0.012 (3)	0.0129 (19)
O4	0.054 (3)	0.035 (2)	0.073 (3)	0.007 (2)	0.002 (2)	0.0077 (19)
O5	0.136 (5)	0.036 (2)	0.051 (2)	0.011 (3)	0.000 (3)	−0.0004 (19)
O6	0.167 (6)	0.036 (2)	0.064 (3)	0.024 (3)	−0.002 (4)	0.006 (2)
O7	0.089 (4)	0.046 (2)	0.045 (2)	0.012 (3)	−0.012 (3)	0.0082 (18)
O8	0.049 (2)	0.036 (2)	0.059 (2)	−0.0048 (19)	0.004 (2)	0.0129 (17)
N1	0.045 (3)	0.053 (3)	0.045 (3)	0.009 (2)	0.001 (2)	−0.010 (2)
N2	0.042 (3)	0.044 (2)	0.044 (3)	−0.002 (2)	−0.003 (2)	0.002 (2)
C1	0.050 (3)	0.038 (3)	0.032 (3)	−0.011 (3)	−0.008 (3)	0.001 (2)
C2	0.059 (4)	0.035 (3)	0.042 (3)	−0.005 (3)	−0.009 (3)	0.003 (2)
C3	0.057 (4)	0.051 (3)	0.048 (3)	−0.003 (3)	−0.004 (3)	0.023 (3)
C4	0.041 (3)	0.046 (3)	0.047 (3)	0.000 (3)	−0.005 (3)	0.008 (2)
C5	0.065 (4)	0.068 (4)	0.044 (3)	0.005 (4)	0.010 (3)	0.008 (3)
C6	0.057 (4)	0.099 (5)	0.038 (3)	0.017 (4)	0.008 (3)	−0.003 (3)
C7	0.029 (3)	0.049 (3)	0.038 (3)	0.005 (3)	−0.004 (2)	−0.006 (2)
C8	0.030 (3)	0.039 (3)	0.040 (3)	−0.001 (2)	−0.010 (2)	0.000 (2)
C9	0.039 (3)	0.036 (3)	0.030 (2)	−0.003 (2)	−0.008 (2)	0.001 (2)
C10	0.139 (8)	0.034 (3)	0.072 (5)	−0.025 (5)	0.013 (6)	−0.007 (3)
C11	0.036 (3)	0.037 (3)	0.048 (3)	0.005 (2)	0.003 (3)	0.001 (2)
C12	0.031 (3)	0.036 (3)	0.039 (3)	−0.003 (2)	−0.007 (2)	−0.001 (2)
C13	0.033 (3)	0.030 (2)	0.033 (2)	−0.009 (2)	−0.006 (2)	0.000 (2)
C14	0.045 (3)	0.034 (3)	0.040 (3)	−0.004 (3)	−0.002 (3)	−0.001 (2)
C15	0.052 (4)	0.048 (3)	0.033 (3)	−0.004 (3)	0.002 (3)	0.000 (2)
C16	0.053 (3)	0.028 (2)	0.035 (3)	−0.004 (3)	−0.007 (3)	0.004 (2)
C17	0.039 (3)	0.030 (3)	0.047 (3)	0.001 (2)	−0.009 (3)	0.002 (2)
C18	0.094 (6)	0.076 (5)	0.061 (4)	0.008 (5)	0.016 (4)	−0.029 (4)
C19	0.085 (5)	0.064 (4)	0.061 (4)	0.001 (4)	0.019 (4)	0.022 (3)
C20	0.081 (6)	0.044 (4)	0.123 (7)	−0.002 (4)	0.005 (6)	−0.018 (4)
C21	0.079 (5)	0.041 (3)	0.035 (3)	0.008 (3)	0.011 (3)	0.006 (2)
C22	0.097 (6)	0.038 (3)	0.057 (4)	0.018 (4)	0.014 (4)	0.010 (3)
C23	0.083 (5)	0.048 (4)	0.051 (4)	0.020 (4)	0.002 (4)	0.023 (3)
C24	0.057 (4)	0.047 (3)	0.040 (3)	0.003 (3)	0.005 (3)	0.008 (3)
C25	0.061 (4)	0.065 (4)	0.053 (4)	0.000 (4)	−0.008 (3)	0.020 (3)
C26	0.061 (4)	0.061 (4)	0.037 (3)	0.003 (3)	−0.006 (3)	0.005 (3)
C27	0.029 (3)	0.038 (3)	0.038 (3)	0.006 (2)	0.000 (2)	0.001 (2)
C28	0.044 (3)	0.038 (3)	0.033 (3)	0.002 (3)	0.012 (3)	0.012 (2)
C29	0.044 (3)	0.033 (3)	0.043 (3)	0.005 (3)	0.010 (3)	0.007 (2)
C30	0.262 (16)	0.037 (4)	0.079 (5)	0.022 (7)	−0.040 (9)	−0.004 (4)
C31	0.042 (3)	0.037 (3)	0.040 (3)	0.001 (3)	0.001 (3)	0.007 (2)
C32	0.036 (3)	0.035 (3)	0.036 (3)	0.002 (2)	0.009 (2)	0.000 (2)
C33	0.045 (3)	0.035 (3)	0.030 (3)	0.006 (3)	0.010 (2)	0.004 (2)
C34	0.062 (4)	0.036 (3)	0.041 (3)	0.001 (3)	0.000 (3)	−0.002 (2)
C35	0.058 (4)	0.052 (3)	0.041 (3)	0.006 (3)	−0.006 (3)	−0.005 (3)
C36	0.056 (4)	0.041 (3)	0.035 (3)	0.010 (3)	0.003 (3)	0.004 (2)
C37	0.049 (3)	0.030 (3)	0.036 (3)	0.001 (2)	0.012 (3)	0.006 (2)
C38	0.094 (6)	0.065 (4)	0.067 (4)	−0.005 (5)	−0.020 (5)	−0.012 (3)
C39	0.119 (7)	0.074 (5)	0.052 (4)	0.034 (5)	−0.026 (5)	0.004 (3)
C40	0.071 (5)	0.043 (3)	0.095 (5)	0.000 (4)	0.004 (5)	−0.001 (3)

### Geometric parameters (Å, °)

**Table:**

O1—C2	1.367 (6)	C15—H15A	0.93
O1—C10	1.413 (9)	C16—C17	1.391 (8)
O2—C1	1.390 (7)	C18—H18A	0.96
O2—C10	1.411 (7)	C18—H18B	0.96
O3—C16	1.370 (6)	C18—H18C	0.96
O3—C19	1.407 (8)	C19—H19A	0.96
O4—C17	1.393 (6)	C19—H19B	0.96
O4—C20	1.408 (8)	C19—H19C	0.96
O5—C21	1.386 (7)	C20—H20A	0.96
O5—C30	1.400 (8)	C20—H20B	0.96
O6—C22	1.389 (7)	C20—H20C	0.96
O6—C30	1.380 (9)	C21—C29	1.382 (7)
O7—C36	1.371 (6)	C21—C22	1.383 (9)
O7—C39	1.428 (8)	C22—C23	1.354 (9)
O8—C37	1.385 (6)	C23—C24	1.399 (8)
O8—C40	1.435 (8)	C23—H23A	0.93
N1—C6	1.430 (8)	C24—C28	1.370 (7)
N1—C18	1.472 (8)	C24—C25	1.529 (8)
N1—C7	1.481 (7)	C25—C26	1.482 (8)
N2—C26	1.466 (7)	C25—H25A	0.97
N2—C27	1.472 (7)	C25—H25B	0.97
N2—C38	1.481 (8)	C26—H26A	0.97
C1—C2	1.364 (7)	C26—H26B	0.97
C1—C9	1.390 (7)	C27—C31	1.515 (7)
C2—C3	1.362 (8)	C27—C28	1.538 (7)
C3—C4	1.406 (8)	C27—H27A	0.98
C3—H3A	0.93	C28—C29	1.410 (8)
C4—C8	1.412 (7)	C29—C33	1.480 (7)
C4—C5	1.486 (8)	C30—H30A	0.97
C5—C6	1.511 (9)	C30—H30B	0.97
C5—H5A	0.97	C31—C32	1.506 (7)
C5—H5B	0.97	C31—H31A	0.97
C6—H6A	0.97	C31—H31B	0.97
C6—H6B	0.97	C32—C37	1.391 (7)
C7—C8	1.523 (7)	C32—C33	1.409 (7)
C7—C11	1.528 (7)	C33—C34	1.400 (8)
C7—H7A	0.98	C34—C35	1.386 (8)
C8—C9	1.405 (7)	C34—H34A	0.93
C9—C13	1.464 (7)	C35—C36	1.394 (8)
C10—H10A	0.97	C35—H35A	0.93
C10—H10B	0.97	C36—C37	1.381 (8)
C11—C12	1.496 (7)	C38—H38A	0.96
C11—H11A	0.97	C38—H38B	0.96
C11—H11B	0.97	C38—H38C	0.96
C12—C17	1.403 (7)	C39—H39A	0.96
C12—C13	1.404 (7)	C39—H39B	0.96
C13—C14	1.406 (7)	C39—H39C	0.96
C14—C15	1.390 (7)	C40—H40A	0.96
C14—H14A	0.93	C40—H40B	0.96
C15—C16	1.362 (7)	C40—H40C	0.96
			
C2—O1—C10	104.8 (4)	H19B—C19—H19C	109.5
C1—O2—C10	104.6 (5)	O4—C20—H20A	109.5
C16—O3—C19	117.8 (5)	O4—C20—H20B	109.5
C17—O4—C20	114.3 (5)	H20A—C20—H20B	109.5
C21—O5—C30	105.2 (5)	O4—C20—H20C	109.5
C22—O6—C30	104.9 (5)	H20A—C20—H20C	109.5
C36—O7—C39	117.2 (5)	H20B—C20—H20C	109.5
C37—O8—C40	111.8 (5)	C29—C21—O5	129.1 (5)
C6—N1—C18	111.2 (5)	C29—C21—C22	122.2 (6)
C6—N1—C7	111.0 (5)	O5—C21—C22	108.6 (5)
C18—N1—C7	110.1 (5)	C23—C22—C21	121.9 (6)
C26—N2—C27	111.1 (4)	C23—C22—O6	128.4 (6)
C26—N2—C38	109.2 (5)	C21—C22—O6	109.7 (6)
C27—N2—C38	110.9 (5)	C22—C23—C24	117.5 (5)
C2—C1—C9	123.8 (5)	C22—C23—H23A	121.3
C2—C1—O2	109.1 (4)	C24—C23—H23A	121.3
C9—C1—O2	126.9 (5)	C28—C24—C23	120.9 (6)
C3—C2—O1	128.0 (5)	C28—C24—C25	120.2 (5)
C3—C2—C1	121.5 (5)	C23—C24—C25	118.9 (5)
O1—C2—C1	110.5 (5)	C26—C25—C24	112.4 (5)
C2—C3—C4	118.2 (5)	C26—C25—H25A	109.1
C2—C3—H3A	120.9	C24—C25—H25A	109.1
C4—C3—H3A	120.9	C26—C25—H25B	109.1
C3—C4—C8	119.4 (5)	C24—C25—H25B	109.1
C3—C4—C5	120.0 (5)	H25A—C25—H25B	107.9
C8—C4—C5	120.6 (5)	N2—C26—C25	110.3 (5)
C4—C5—C6	111.7 (5)	N2—C26—H26A	109.6
C4—C5—H5A	109.3	C25—C26—H26A	109.6
C6—C5—H5A	109.3	N2—C26—H26B	109.6
C4—C5—H5B	109.3	C25—C26—H26B	109.6
C6—C5—H5B	109.3	H26A—C26—H26B	108.1
H5A—C5—H5B	107.9	N2—C27—C31	111.0 (4)
N1—C6—C5	111.1 (5)	N2—C27—C28	111.1 (4)
N1—C6—H6A	109.4	C31—C27—C28	109.5 (4)
C5—C6—H6A	109.4	N2—C27—H27A	108.4
N1—C6—H6B	109.4	C31—C27—H27A	108.4
C5—C6—H6B	109.4	C28—C27—H27A	108.4
H6A—C6—H6B	108	C24—C28—C29	122.0 (5)
N1—C7—C8	112.0 (5)	C24—C28—C27	121.6 (5)
N1—C7—C11	111.1 (4)	C29—C28—C27	116.4 (4)
C8—C7—C11	109.2 (4)	C21—C29—C28	115.5 (5)
N1—C7—H7A	108.1	C21—C29—C33	123.2 (5)
C8—C7—H7A	108.1	C28—C29—C33	121.3 (4)
C11—C7—H7A	108.1	O6—C30—O5	111.1 (5)
C9—C8—C4	122.1 (5)	O6—C30—H30A	109.4
C9—C8—C7	117.3 (5)	O5—C30—H30A	109.4
C4—C8—C7	120.7 (5)	O6—C30—H30B	109.4
C1—C9—C8	114.9 (5)	O5—C30—H30B	109.4
C1—C9—C13	124.5 (5)	H30A—C30—H30B	108
C8—C9—C13	120.6 (4)	C32—C31—C27	110.8 (4)
O2—C10—O1	109.0 (5)	C32—C31—H31A	109.5
O2—C10—H10A	109.9	C27—C31—H31A	109.5
O1—C10—H10A	109.9	C32—C31—H31B	109.5
O2—C10—H10B	109.9	C27—C31—H31B	109.5
O1—C10—H10B	109.9	H31A—C31—H31B	108.1
H10A—C10—H10B	108.3	C37—C32—C33	119.2 (5)
C12—C11—C7	110.4 (4)	C37—C32—C31	121.5 (5)
C12—C11—H11A	109.6	C33—C32—C31	119.3 (5)
C7—C11—H11A	109.6	C34—C33—C32	118.7 (5)
C12—C11—H11B	109.6	C34—C33—C29	123.7 (5)
C7—C11—H11B	109.6	C32—C33—C29	117.6 (5)
H11A—C11—H11B	108.1	C35—C34—C33	121.3 (5)
C17—C12—C13	118.9 (5)	C35—C34—H34A	119.4
C17—C12—C11	121.0 (5)	C33—C34—H34A	119.4
C13—C12—C11	120.2 (5)	C34—C35—C36	119.7 (6)
C14—C13—C12	118.8 (5)	C34—C35—H35A	120.1
C14—C13—C9	123.2 (5)	C36—C35—H35A	120.1
C12—C13—C9	118.0 (5)	O7—C36—C37	116.0 (5)
C15—C14—C13	120.7 (5)	O7—C36—C35	124.5 (5)
C15—C14—H14A	119.7	C37—C36—C35	119.5 (5)
C13—C14—H14A	119.7	C36—C37—O8	119.3 (5)
C16—C15—C14	120.8 (5)	C36—C37—C32	121.6 (5)
C16—C15—H15A	119.6	O8—C37—C32	119.1 (5)
C14—C15—H15A	119.6	N2—C38—H38A	109.5
C15—C16—O3	125.0 (5)	N2—C38—H38B	109.5
C15—C16—C17	119.6 (5)	H38A—C38—H38B	109.5
O3—C16—C17	115.4 (5)	N2—C38—H38C	109.5
O4—C17—C16	120.3 (5)	H38A—C38—H38C	109.5
O4—C17—C12	118.4 (5)	H38B—C38—H38C	109.5
C16—C17—C12	121.3 (5)	O7—C39—H39A	109.5
N1—C18—H18A	109.5	O7—C39—H39B	109.5
N1—C18—H18B	109.5	H39A—C39—H39B	109.5
H18A—C18—H18B	109.5	O7—C39—H39C	109.5
N1—C18—H18C	109.5	H39A—C39—H39C	109.5
H18A—C18—H18C	109.5	H39B—C39—H39C	109.5
H18B—C18—H18C	109.5	O8—C40—H40A	109.5
O3—C19—H19A	109.5	O8—C40—H40B	109.5
O3—C19—H19B	109.5	H40A—C40—H40B	109.5
H19A—C19—H19B	109.5	O8—C40—H40C	109.5
O3—C19—H19C	109.5	H40A—C40—H40C	109.5
H19A—C19—H19C	109.5	H40B—C40—H40C	109.5
			
C10—O2—C1—C2	−8.6 (8)	C30—O5—C21—C29	−174.6 (9)
C10—O2—C1—C9	176.2 (7)	C30—O5—C21—C22	2.0 (11)
C10—O1—C2—C3	−172.4 (7)	C29—C21—C22—C23	−1.8 (13)
C10—O1—C2—C1	7.9 (8)	O5—C21—C22—C23	−178.7 (8)
C9—C1—C2—C3	−3.8 (10)	C29—C21—C22—O6	179.1 (7)
O2—C1—C2—C3	−179.3 (6)	O5—C21—C22—O6	2.2 (10)
C9—C1—C2—O1	175.9 (6)	C30—O6—C22—C23	175.5 (11)
O2—C1—C2—O1	0.4 (7)	C30—O6—C22—C21	−5.4 (11)
O1—C2—C3—C4	−179.5 (6)	C21—C22—C23—C24	1.5 (13)
C1—C2—C3—C4	0.2 (10)	O6—C22—C23—C24	−179.6 (8)
C2—C3—C4—C8	2.3 (9)	C22—C23—C24—C28	−0.5 (11)
C2—C3—C4—C5	−179.2 (6)	C22—C23—C24—C25	178.4 (7)
C3—C4—C5—C6	163.8 (6)	C28—C24—C25—C26	−17.2 (9)
C8—C4—C5—C6	−17.8 (8)	C23—C24—C25—C26	163.9 (6)
C18—N1—C6—C5	169.7 (6)	C27—N2—C26—C25	−67.8 (7)
C7—N1—C6—C5	−67.3 (7)	C38—N2—C26—C25	169.5 (6)
C4—C5—C6—N1	50.5 (8)	C24—C25—C26—N2	48.9 (8)
C6—N1—C7—C8	48.1 (6)	C26—N2—C27—C31	172.1 (5)
C18—N1—C7—C8	171.7 (5)	C38—N2—C27—C31	−66.2 (6)
C6—N1—C7—C11	170.5 (5)	C26—N2—C27—C28	50.0 (6)
C18—N1—C7—C11	−65.9 (6)	C38—N2—C27—C28	171.7 (5)
C3—C4—C8—C9	−1.3 (9)	C23—C24—C28—C29	−0.3 (9)
C5—C4—C8—C9	−179.8 (6)	C25—C24—C28—C29	−179.1 (6)
C3—C4—C8—C7	179.9 (5)	C23—C24—C28—C27	−179.4 (6)
C5—C4—C8—C7	1.4 (8)	C25—C24—C28—C27	1.8 (9)
N1—C7—C8—C9	165.5 (5)	N2—C27—C28—C24	−17.9 (7)
C11—C7—C8—C9	42.0 (7)	C31—C27—C28—C24	−140.8 (5)
N1—C7—C8—C4	−15.6 (7)	N2—C27—C28—C29	163.0 (5)
C11—C7—C8—C4	−139.1 (5)	C31—C27—C28—C29	40.0 (7)
C2—C1—C9—C8	4.6 (9)	O5—C21—C29—C28	177.2 (7)
O2—C1—C9—C8	179.2 (5)	C22—C21—C29—C28	1.0 (10)
C2—C1—C9—C13	−173.5 (5)	O5—C21—C29—C33	−1.6 (12)
O2—C1—C9—C13	1.1 (10)	C22—C21—C29—C33	−177.8 (7)
C4—C8—C9—C1	−2.0 (8)	C24—C28—C29—C21	0.0 (9)
C7—C8—C9—C1	176.8 (5)	C27—C28—C29—C21	179.2 (6)
C4—C8—C9—C13	176.2 (5)	C24—C28—C29—C33	178.8 (6)
C7—C8—C9—C13	−4.9 (7)	C27—C28—C29—C33	−2.0 (8)
C1—O2—C10—O1	13.5 (8)	C22—O6—C30—O5	6.8 (14)
C2—O1—C10—O2	−13.3 (8)	C21—O5—C30—O6	−5.5 (14)
N1—C7—C11—C12	−179.0 (4)	N2—C27—C31—C32	−179.4 (4)
C8—C7—C11—C12	−55.0 (6)	C28—C27—C31—C32	−56.4 (6)
C7—C11—C12—C17	−146.2 (5)	C27—C31—C32—C37	−142.4 (5)
C7—C11—C12—C13	34.3 (7)	C27—C31—C32—C33	37.6 (7)
C17—C12—C13—C14	2.2 (7)	C37—C32—C33—C34	1.6 (8)
C11—C12—C13—C14	−178.3 (5)	C31—C32—C33—C34	−178.4 (5)
C17—C12—C13—C9	−176.2 (5)	C37—C32—C33—C29	−179.1 (5)
C11—C12—C13—C9	3.3 (7)	C31—C32—C33—C29	0.9 (7)
C1—C9—C13—C14	−19.7 (8)	C21—C29—C33—C34	−22.0 (9)
C8—C9—C13—C14	162.3 (5)	C28—C29—C33—C34	159.3 (6)
C1—C9—C13—C12	158.6 (6)	C21—C29—C33—C32	158.8 (6)
C8—C9—C13—C12	−19.4 (7)	C28—C29—C33—C32	−19.9 (8)
C12—C13—C14—C15	−2.0 (8)	C32—C33—C34—C35	−3.3 (9)
C9—C13—C14—C15	176.3 (5)	C29—C33—C34—C35	177.6 (6)
C13—C14—C15—C16	0.6 (9)	C33—C34—C35—C36	2.1 (10)
C14—C15—C16—O3	−178.5 (5)	C39—O7—C36—C37	−176.0 (6)
C14—C15—C16—C17	0.5 (9)	C39—O7—C36—C35	3.4 (10)
C19—O3—C16—C15	−1.5 (9)	C34—C35—C36—O7	−178.5 (6)
C19—O3—C16—C17	179.5 (6)	C34—C35—C36—C37	0.8 (10)
C20—O4—C17—C16	−74.0 (7)	O7—C36—C37—O8	−0.1 (8)
C20—O4—C17—C12	107.8 (6)	C35—C36—C37—O8	−179.5 (6)
C15—C16—C17—O4	−178.4 (5)	O7—C36—C37—C32	177.0 (5)
O3—C16—C17—O4	0.6 (8)	C35—C36—C37—C32	−2.4 (9)
C15—C16—C17—C12	−0.3 (8)	C40—O8—C37—C36	−80.1 (7)
O3—C16—C17—C12	178.8 (5)	C40—O8—C37—C32	102.8 (6)
C13—C12—C17—O4	177.1 (5)	C33—C32—C37—C36	1.2 (8)
C11—C12—C17—O4	−2.4 (8)	C31—C32—C37—C36	−178.8 (5)
C13—C12—C17—C16	−1.1 (8)	C33—C32—C37—O8	178.2 (5)
C11—C12—C17—C16	179.4 (5)	C31—C32—C37—O8	−1.8 (8)
